# Endodontic Management of Teeth Juxtaposed to Haemangioma

**DOI:** 10.1155/2017/9791851

**Published:** 2017-03-20

**Authors:** Rekha Chandra Mani, Anchu Rachel Thomas, Premkumar Elavarasu, Vijay Venkatesh

**Affiliations:** ^1^SRM University, Kattankulathur, India; ^2^Department of Conservative Dentistry, SRM University, Kattankulathur, India

## Abstract

Vascular anomalies are localized defects in the vasculature that may or may not be present at birth. There are many types of vascular anomalies with different aetiology and clinical picture and, therefore, require the combined expertise of medical, radiological, and surgical specialities for its diagnosis and management. The term “haemangioma” is used as a common generic label to incorporate all types of vascular malformations. In this report, we describe a case of two maxillary premolars, requiring endodontic therapy, in close proximity to a haemangioma. The challenges encountered in the diagnosis and management of the case are discussed.

## 1. Introduction

Haemangiomas are developmental malformations of the blood vessels and not typically tumours. They can be congenital or acquired [1] and are characterised by endothelial cell hyperplasia which undergoes involution in most cases. These lesions show a female predilection with a ratio of 3 : 1 and most commonly affect the head and neck region [2].

Haemangiomas never turn malignant and are usually present from birth. Histologically, they show numerous nonlobular proliferations of blood vessels with chronic inflammatory stroma [2]. Radiologically, intraosseous haemangiomas reveal bony expansion, whereas the nonintraosseous types reveal a radiolucent lesion with distinct margins and honey comb pattern [2]. Treatment options for haemangioma include wait and watch approach, topical and systemic steroids, injection of a sclerosing agent (sclerotherapy), laser therapy, cautery, and surgical excision [3].

This article reports a case of successful endodontic treatment of two maxillary premolars in juxtaposition to a haemangioma. This case could have easily been mistaken for a dentoalveolar abscess, as teeth requiring endodontic treatment were present in the vicinity of the lesion.

## 2. Case Presentation

**Figure 1:**
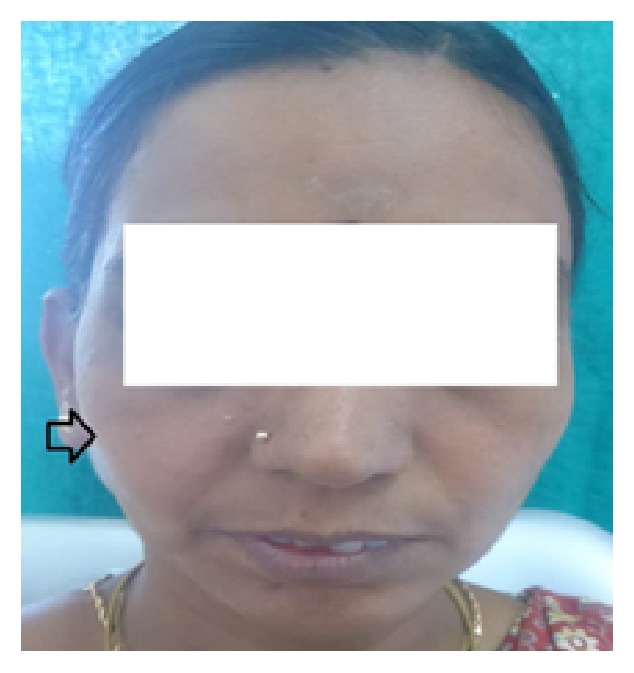
Extraoral swelling present on the right side infraorbital region.

A forty-five-year-old female reported to the Department of Conservative Dentistry and Endodontics, with the chief complaint of pain in #14 and #15 and extra oral swelling on the right side infraorbital region (Figure 1). History revealed that the swelling was present for more than a year. It was asymptomatic and slow growing in nature. Patient had experienced occasional hypersensitivity with food impaction in #14 and #15 for the past few months. But a severe pain of sudden onset led her to seek dental assistance. General physical examination revealed that patient was normally built for her age. There was no defect in stature or gait. Her medical and family histories were noncontributory.

**Figure 2:**
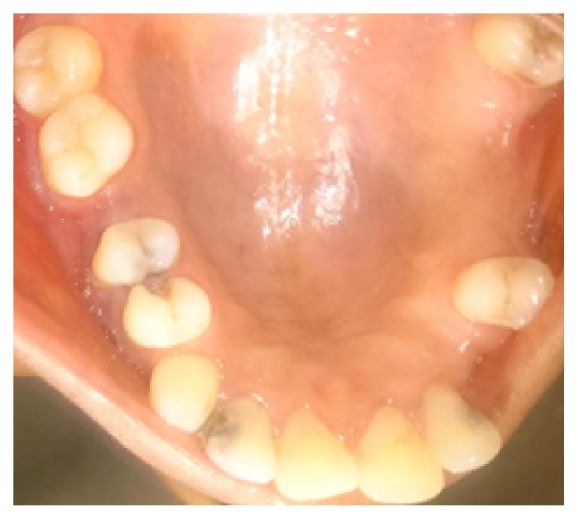
Intraoral image of #14 and #15 region shows no obliteration of buccal vestibule.

**Figure 3:**
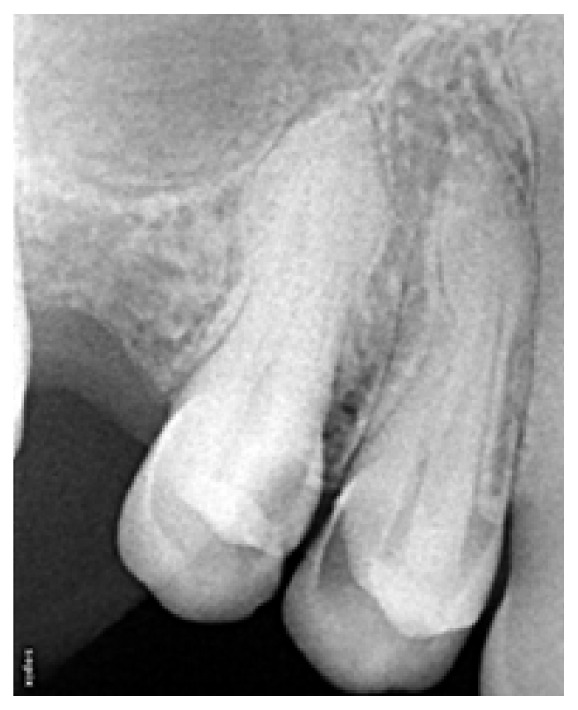
Intraoral periapical radiograph of #14 and #15 region shows coronal radiolucency in 14 (distal) and 15 (mesial). The radiolucency approximates to the pulp. There is also evidence of PDL widening in both teeth.

**Figure 4:**
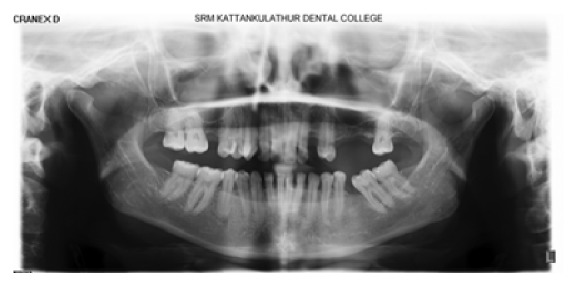
Orthopantomograph shows coronal radiolucency in #14 and #15 with multiple edentulous spaces. No periapical radiolucencies were evident.

Extraoral examination revealed a diffuse swelling extending from the inferior border of orbit to the level of occlusal plane. The overlying skin was normal in colour. On palpation, swelling was soft and compressible in nature. Intraoral examination revealed carious involvement in 14 and 15 with both teeth exhibiting tenderness to percussion. There was no obliteration of the buccal vestibule in spite of the presence of extraoral swelling (Figure 2). Intraoral periapical radiographs (Figure 3) revealed coronal radiolucency extending into the pulp accompanied by widening of the periodontal ligament space in both #14 and #15. Orthopantomographs (Figure 4) were advised to investigate the radiographic changes in relation to the extraoral swelling, but in vain. Both #14 and #15 responded positively to electric and cold pulp testing. Consequently, it was concluded that the swelling may not be of dental origin. Complete blood and urine investigations were performed which ruled out systemic conditions like diabetes mellitus, HIV, HSs, tuberculosis, or leukemic enlargement. Thereby any possibility of an infectious enlargement was dismissed.

**Figure 5:**
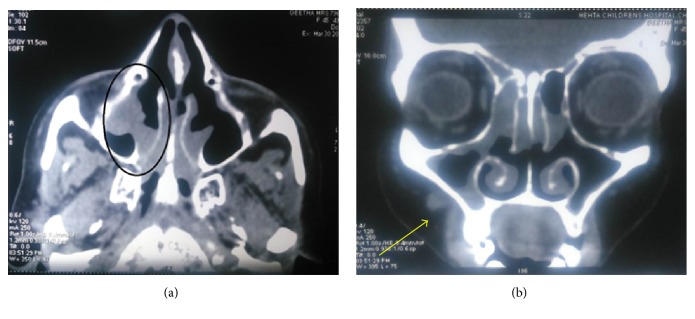
(a) CT image of the lesion: right posterior maxillary region showing irregular soft tissue growth. (b) Coronal CT view in which the location of the haemangioma is being shown by an arrow.

The patient was referred to Department of General Medicine for opinion regarding the extraoral swelling. CT scans were taken which revealed an irregular soft tissue mass measuring 18 × 16 mm in the subcutaneous plane of the posterior maxillary region (Figures 5(a) and 5(b)) on the right side. The lesion appeared separate from the maxillary antrum and there was no bony expansion. Duplex ultrasonography confirmed the presence of a single feeding vessel into the lesion and the lesion was diagnosed as haemangioma. Patient was referred to the vascular surgeon who was further briefed about the need for endodontic therapy. Patient was advised sclerotherapy under Duplex ultrasonographic guidance. 1 mL of foam 3% polidocanol was injected intralesionally and compression bandage was applied. A one-month follow-up CT showed a significant reduction in the size of the lesion (18 × 12 mm) and consent was given to proceed with endodontic treatment.

**Figure 6:**
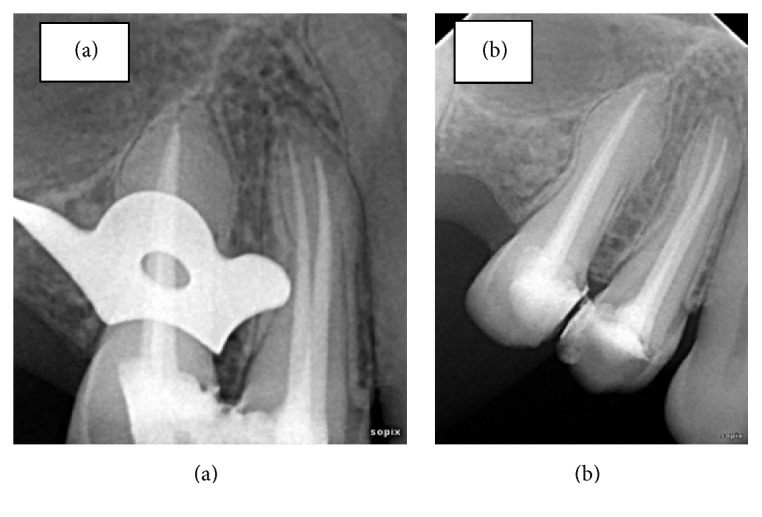
(a) and (b) showing obturation and 6-month follow-up radiographs, respectively.

Local anaesthetic (Lignox 2%A) was administered via infiltration. Access opening was done in #14 and #15, under rubber dam isolation. Working length determination was done using an electronic apex locator (J Morita, USA, Inc.) and later confirmed radiographically to circumvent the risk of overinstrumentation. Canals were irrigated with 3% sodium hypochlorite. Root canals were cleaned and shaped with Protaper files (Dentsply-maillefer, Ballaigues) up to size F2 and obturated in a single visit (Figure 6(a)). Patient was reviewed after 1 week during which she was asymptomatic and postendodontic restoration was done with light cure composite resin (3M ESPE, USA). Patient continued to remain asymptomatic during follow-ups done at 3 and 6 months (Figure 6(b)) and was under her regular sclerotherapy sessions. Informed written consent was obtained from the patient with regards to the publication of her extraoral, intraoral, and radiographic images.

## 3. Discussion

Untreated pulpal inflammation causes apical periodontitis, the earliest periapical change characterized by thickening of the ligament at the root apex. Further invasion of pathogens into the periapical area results in periapical abscess/cyst formation. Acute periapical abscesses do not show radiographic signs of its presence except for the thickening of periodontal ligament space. However, chronic abscesses exhibit an ill-defined radiolucency at the apex of the tooth. If the disease progression is not controlled, osteomyelitis, periostitis, and finally cellulitis develop [4].

In this case, haemangioma was present in the close vicinity of teeth with apical periodontitis. The clinical signs of haemangioma were not much evident, except for a mild swelling. Adhering to standard diagnostic protocol and clinical investigations aided in ruling out a dental cause for the swelling. Uncontrolled spread of infection could lead to entry of pathogens into the site of haemangioma. Hence, control of infection either by extraction or endodontic therapy was mandatory. In this case, endodontic therapy was preferred as the presence of haemangioma complicated any form of surgical intervention.

Sclerotherapy was opted for this case because it is considered the most effective modality for management of localized vascular lesions [5]. Foam polidocanol when injected intravascularly displaces blood column, adheres to the endothelium, and causes spasm [6]. Foam polidocanol has greater durability and ability to penetrate the collateral vessels than its liquid counterpart [7]. Another advantage is that foam is highly echogenic facilitating sclerotherapy under ultrasound guidance wherein the safety of sclerosis is increased [8].

In this case, the following precautions were taken during treatment to prevent entry of endodontic irrigants, instruments, and obturation materials beyond the apex of the tooth. Initially, lignocaine containing 1 : 80000 adrenaline was administered via infiltration. Rubber dam placement prevented soft tissue laceration during instrumentation. Working length was initially determined using apex locators followed by confirmatory radiographs to prevent the likelihood of instrumentation beyond the root apex. 3% sodium hypochlorite solution was used for irrigation during the entire cleaning and shaping procedure. Use of sodium hypochlorite followed by placement of calcium hydroxide intracanal medicament has shown to minimize the risk of excess bleed during endodontic therapy [9]. All root canal procedures were terminated 2 mm short of the radiographic apex, leaving behind an apical pulp stump to prevent untoward extrusion of irritating irrigants and filling materials beyond the apex. Clinical and biologic evidence also suggest a favourable outcome for vital teeth when the root canal procedures were terminated 2 to 3 mm short of the radiographic apex [10]. For posttreatment pain management, NSAIDS were administered following discussion with the concerned vascular surgeon.

## 4. Conclusion

This article emphasizes the importance of recording a detailed case history and investigations in the diagnosis of pathology not associated with teeth. In the present case, the extraoral swelling was closely mimicking a dentoalveolar abscess as teeth requiring endodontic therapy were present in relation to the swelling. Faulty diagnosis and treatment initiation without precautionary measures might have led to an untoward entry into the site of haemangioma.
